# Caspase-Cleaved Keratin 18 Measurements Identified Ongoing Liver Injury after Bariatric Surgery

**DOI:** 10.3390/jcm10061233

**Published:** 2021-03-16

**Authors:** Felix Hempel, Martin Roderfeld, Lucas John Müntnich, Jens Albrecht, Ziya Oruc, Borros Arneth, Thomas Karrasch, Jörn Pons-Kühnemann, Winfried Padberg, Harald Renz, Andreas Schäffler, Elke Roeb

**Affiliations:** 1 Department of Gastroenterology, Justus Liebig University, D-35392 Giessen, Germany; felix.hempel@med.jlug.de (F.H.); martin.roderfeld@innere.med.uni-giessen.de (M.R.); lucas.j.muentnich@med.uni-giessen.de (L.J.M.); 2 Department of General, Visceral, Thoracic, Transplantation, and Pediatric Surgery, Justus Liebig University, D-35392 Giessen, Germany; j.albrecht@azhm.de (J.A.); z.oruc@web.de (Z.O.); winfried.padberg@chiru.med.uni-giessen.de (W.P.); 3 Department for Bariatric Surgery, Asklepios Hospital, D-35423 Lich, Germany; 4 Institute of Laboratory Medicine, Pathobiochemistry and Molecular Diagnostics, Justus Liebig University, D-35392 Giessen, Germany; borros.arneth@klinchemie.med.uni-giessen.de (B.A.); renzh@med.uni-marburg.de (H.R.); 5 The German Lung Center (DZL) and the Universities of Giessen and Marburg Lung Center (UGMLC), D-35392 Giessen, Germany; 6 Department of Internal Medicine III, Justus Liebig University, D-35392 Giessen, Germany; thomas.karrasch@innere.med.uni-giessen.de (T.K.); andreas.schaeffler@innere.med.uni-giessen.de (A.S.); 7 Institute of Medical Informatics, Justus Liebig University, D-35392 Giessen, Germany; Joern.Pons@informatik.med.uni-giessen.de

**Keywords:** non-alcoholic fatty liver disease, NAFLD, NASH, keratin 18, cytokeratin 18, M30, gastric bypass, non-invasive biomarkers

## Abstract

Bariatric surgery has emerged as an effective treatment option in morbidly obese patients with non-alcoholic fatty liver disease (NAFLD). However, worsening or new onset of non-alcoholic steatohepatitis (NASH) and fibrosis have been observed. Caspase-cleaved keratin 18 (ccK18) has been established as a marker of hepatocyte apoptosis, a key event in NASH development. Thus, ccK18 measurements might be feasible to monitor bariatric surgery patients. Clinical data and laboratory parameters were collected from 39 patients undergoing laparoscopic Roux-en-Y gastric bypass at six timepoints, prior to surgery until one year after the procedure. ccK18 levels were measured and a high-throughput analysis of serum adipokines and cytokines was carried out. Half of the cohort’s patients (20/39) presented with ccK18 levels indicative of progressed liver disease. 21% had a NAFLD-fibrosis score greater than 0.676, suggesting significant fibrosis. One year after surgery, a mean weight loss of 36.87% was achieved. Six and twelve months after surgery, ccK18 fragments were significantly reduced compared to preoperative levels (*p* < 0.001). Yet nine patients did not show a decline in ccK18 levels ≥ 10% within one year postoperatively, which was considered a response to treatment. While no significant differences in laboratory parameters or ccK18 could be observed, they presented with a greater expression of leptin and fibrinogen before surgery. Consecutive ccK18 measurements monitored the resolution of NAFLD and identified non-responders to bariatric surgery with ongoing liver injury. Further studies are needed to elicit the pathological mechanisms in non-responders and study the potential of adipokines as prognostic markers.

## 1. Introduction

Non-alcoholic fatty liver disease (NAFLD) is considered the hepatic manifestation of metabolic syndrome. It comprises a spectrum of diseases from simple steatosis to nonalcoholic steatohepatitis (NASH), cirrhosis of the liver and complications such as hepatocellular carcinoma (HCC) [1,2]. NAFLD is extremely prevalent and its importance in the etiology of liver failure, HCC, and liver transplantation is increasing rapidly [3,4]. Although numerous studies have delineated the complex pathophysiological mechanisms of NAFLD in past decades, no approved drug treatment is available yet [5]. Established therapeutic concepts remain limited to the treatment of underlying metabolic dysregulations. Herein, bariatric surgery has emerged as an effective intervention in morbidly obese patients [6,7,8]. International guidelines and guidance statements recommend considering bariatric surgery, if lifestyle interventions fail [9,10,11,12].

Conversely, NAFLD is particularly common among patients undergoing bariatric surgery [13,14,15]. Thus, hepatologic counseling is needed for timely diagnosis, patient monitoring, and treatment optimization. This seems especially important in the context of the worsening or new onset of NAFLD, which has been observed in bariatric surgery trials. A recent meta-analysis found such a response in 5%–20% of patients [8]. Yet the concepts of these clinical trials rely on repeated liver biopsies. Although currently considered the gold standard in the diagnosis of NAFLD, risks and cost of the procedure and shortcomings of the technique itself (e.g., sampling error and interrater variability) forbid a widespread application in everyday clinical practice. Therefore, non-invasive biomarkers are needed to enable hepatologic surveillance, especially in bariatric surgery patients.

Among many potential candidates, caspase-cleaved keratin 18 (ccK18) is deemed a promising novel biomarker. During hepatocyte apoptosis, ccK18 fragments enter the bloodstream, allowing their detection by the M30 enzyme-linked immunosorbent assay (ELISA) [16,17]. Thus, elevated ccK18 levels were linked to chronic liver disease [18,19] and–as it became evident that apoptotic hepatocytes are a major pathophysiological feature of NAFLD–ccK18 was studied extensively as a non-invasive biomarker for NAFLD [20,21,22,23,24,25,26,27]. Thus far, a fair diagnostic accuracy has been demonstrated and ccK18, alone or in combined biomarker panels, is expected to enter clinical practice soon [28,29]. The marker’s responsiveness following an intervention has been shown both after pharmacologic interventions and diet-induced weight loss [30,31,32]. In conclusion, ccK18 measurements might be a feasible way to monitor the disease progression in bariatric surgery patients.

We aimed to evaluate the use of ccK18 as a biomarker for NAFLD in the follow-up of a cohort undergoing bariatric surgery. We aimed to elucidate (1) the prevalence of NAFLD, based on ccK18 levels and established fibrosis scores, before and after surgery, (2) the natural history of ccK18 levels after bariatric surgery, and (3) whether inconsistencies in the response to bariatric surgery occur and if the addition of ccK18 to a standard follow-up laboratory panel might enable the prediction thereof.

## 2. Materials and Methods

### 2.1. Patients

Clinical data and serum samples were collected from consecutive patients undergoing laparoscopic Roux-en-Y gastric bypass (RYGB) at the Obesity Center at the University of Giessen, Germany. The decision for bariatric surgery was made in accordance with current guidelines, requiring a body mass index (BMI) > 40 kg/m^2^ (or 35 kg/m^2^ and type 2 diabetes mellitus), the failure of conservative weight loss efforts, and the absence of contraindications. Prior bariatric surgery led to the exclusion of patients from the current study. A medical history was obtained and patients were examined. Informed consent was obtained from all patients. The study was approved by the local ethics committee at the Justus Liebig University (AZ 60/16) and conducted in accordance with the declaration of Helsinki.

### 2.2. Surgery

The RYGB procedure was carried out by a single experienced surgeon at a single tertiary care center, embedded in a multidisciplinary treatment regimen. Gastric bypass and simultaneous fundectomy followed by a circular gastrojejunostomy were performed. An 8–10 cm pouch was created, and lengths of the biliopancreatic and alimentary limb were set to 70–90 cm and 140–160 cm, respectively.

### 2.3. Data Acquisition

Data were collected at six time points: several days prior to the surgery, 1–3 days after the surgery, 1, 3, 6 and 12 months postoperatively. Each time, anthropometric measurements and a routine laboratory panel were performed. Additional serum samples for the quantification of ccK18 levels were either drawn in clinic visits or provided by the Institute of Laboratory Medicine. At two timepoints, prior to the surgical procedure and six months after, blood samples were collected after a standard meal in addition to fasting samples.

### 2.4. Quantification of Serum ccK18 Levels

ccK18 levels were measured utilizing the Peviva^®^ M30 Apoptosense^®^ ELISA Kit (TECOmedical, Sissach, Switzerland). All measurements were performed in duplicate, according to the manufacturer’s instructions. Concentrations were determined using an Infinite^®^ 200 Pro microplate reader with Magellan™ data analysis software (TECAN, Männedorf, Switzerland), applying a four-parameter logistic regression.

### 2.5. Definition of Responders and Non-Responders to Bariatric Surgery

A reduction of ccK18 levels ≥10% one year after the RYGB procedure when compared to preoperative levels was defined as response to bariatric surgery. Responders and non-responders were compared during data analysis after the allocation was performed based on serum ccK18 levels.

### 2.6. Proteome Profiling

Human Adipokine and Cytokine Array Kits (ARY024 and ARY005B, R&D Systems, Minneapolis, MN, USA) were used to test pooled serum samples of responders and non-responders to bariatric surgery (*n* = 9 per group) before and one year after surgery. Experiments were performed and mean grey values retrieved as described earlier [33]. For each analyte, the expression relative to positive control dots as well as the difference of relative expressions preoperatively and one year postoperatively were calculated.

### 2.7. Statistical Analysis

Data collection, calculation of scores, and descriptive statistics were performed using SPSS Statistics, version 25 (IBM Corp., Armonk, NY, USA). Further statistical analysis was performed using Prism 8 for macOS, version 8.4.3 (GraphPad software, La Jolla, CA, USA). A fitted mixed effect model, accounting for the repeated measures design, using the Geisser-Greenhouse correction, was applied to evaluate the changes in readout parameters in all patients over time. For the comparison of responders and non-responders to bariatric surgery, two-way ANOVA was used for each individual timepoint. Sidak’s multiple comparisons test was applied in all cases. The significance level α was set to 0.05.

## 3. Results

### 3.1. Preoperative Assessment of the Study Population

39 patients were included in the present study. Table 1 summarizes the baseline patient characteristics. The majority of participants was female, ranging from age 23 to 60. All of them were morbidly obese (BMI > 40 kg/m^2^) with a mean BMI of 51.94 kg/m^2^ before surgery. None of the patients had an established diagnosis of chronic liver disease. However, considering the additional moderate to high prevalence of diabetes mellitus or preconditions of impaired glucose tolerance, hyperlipidemia, and elevated liver transaminases (Table 1), our study population comprises major risk factors, especially for NAFLD. In fact, about half of the patients (20/39) presented with ccK18 levels that have been proposed indicative of progressed liver disease (>200 U/l) [18,24,25]. In order to further characterize our study population and evaluate the likelihood of advanced fibrosis, we applied established non-invasive biomarkers of liver fibrosis. While few or no patients exceeded the cutoff for the APRI or FIB-4 index [34,35], about 21% of the participants had a NAFLD-fibrosis score (NFS) greater than 0.676, suggesting significant (stage 3–4) fibrosis [36].

### 3.2. Roux-en-Y Gastric Bypass Induced Severe Weight Loss and Improved the Patient’s Metabolic State

**Figure 1:**
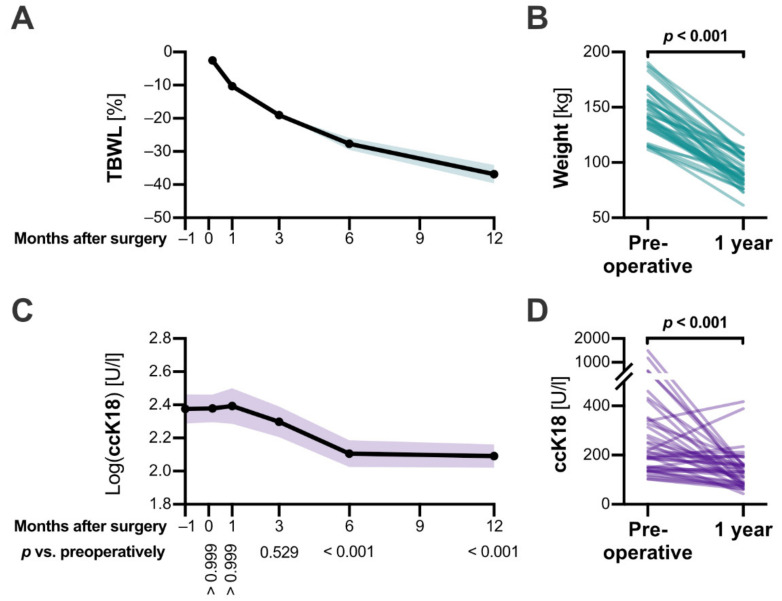
Roux-en-Y gastric bypass led to decreased body weight and caspase-cleaved keratin 18 levels. (**A**) Following the bariatric surgery, patients consistently lost weight with a mean total body weight loss (TBWL) of 37% after one year. Mean (line) and 95% confidence interval (colored area) are shown. (**B**) The reduction in body weight, affecting all included patients, was highly significant 12 months postoperatively when compared to preoperative levels (*p* < 0.001). (**C**) Caspase-cleaved keratin 18 (ccK18) fragments were measured utilizing a M30 enzyme-linked immunosorbent assay. Serum levels decreased significantly within 6 months after the procedure. Mean (line) and 95% confidence interval (colored area) are shown. (**D**) The individual course of ccK18 levels was, however, heterogenous among our cohort. A fitted mixed effect model was applied.

Following bariatric surgery, all patients showed a steady loss of body weight (Figure 1A,B). One year after surgery, a mean total body weight loss (TBWL) of 36.87% (95% CI: 34.16%–39.57%) and an excessive weight loss (EWL) of 71.89% (66.87%–76.91%) were achieved (Table 1). The mean BMI was 32.64 (95% CI 31.38–34.1). The success of bariatric surgery was consistent throughout the entire patient collective, and the minimum TBWL was 17.88%. Metabolic parameters, such as HbA1c, LDL-cholesterol, and serum triglycerides significantly improved as consequence of the intervention (Table 1).

### 3.3. ccK18 Levels Decreased within Six Months after Surgery

To monitor the development of ccK18 levels after RYGB, we collected additional blood samples at six time points–several days before until one year after the surgery. Fasting blood samples were not required to obtain reliable ccK18 results. The coefficient of variation, comparing fasting and non-fasting blood samples at two time points (8.15% and 8.68%), undercut the inter-assay variability given by the manufacturer (<10%, see Supplementary Figure S1). Interestingly, the ccK18 levels we obtained followed a lognormal distribution, thus their respective logarithms were used for further analysis. During the first month after bariatric surgery, no alterations in mean ccK18 levels could be observed (Figure 1C). At both six and twelve months, ccK18 fragments were significantly reduced compared to preoperative levels (*p* < 0.001). Thereby, the natural history of ccK18 after bariatric surgery was distinct from other parameters of liver cell damage, such as alanine-aminotransferase, aspartate-aminotransferase, or gamma-glutamyl-transferase (GGT), which showed an initial increase subsequent to surgery, followed by a quick decline (Figure S2). One year after RYGB, only five out of the 39 patients had ccK18 levels greater than 200 U/l (Table 1).

### 3.4. The Response to Bariatric Surgery Was Inconsistent among Patients

**Figure 2:**
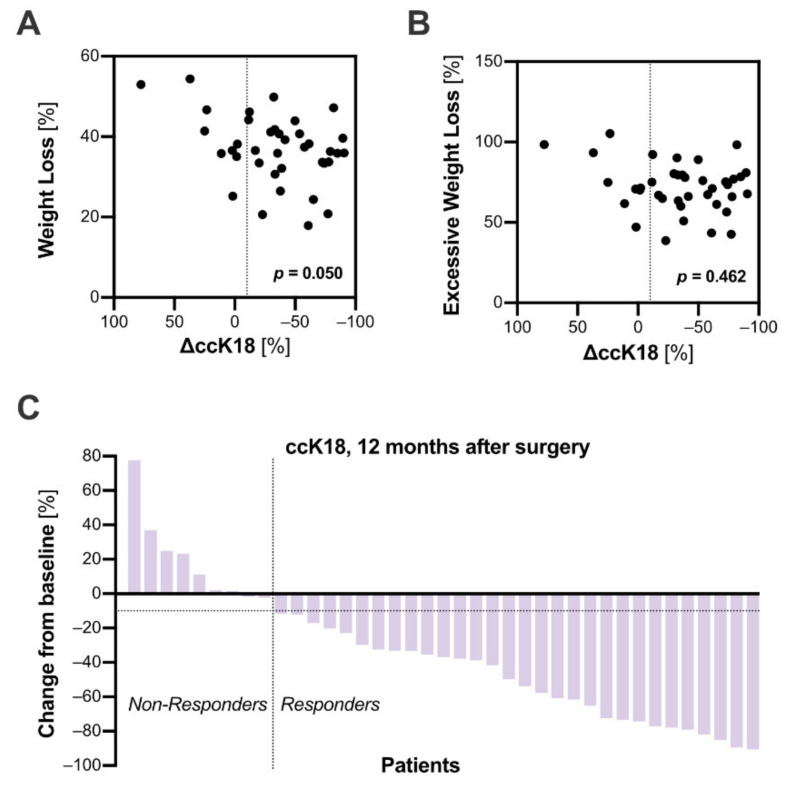
Consecutive ccK18 measurements identified non-responders to bariatric surgery. The individual changes in ccK18 levels during the one-year follow up (∆ccK18) were neither associated with (**A**) total body weight loss (r = 0.32), nor with (**B**) excessive weight loss (r = 0.12). Excessive weight loss was calculated relative to a body mass index of 25. Spearman’s correlation coefficient was applied. Panel (**C**) shows a waterfall plot, depicting the individual change in ccK18 levels 12 months after surgery, compared to preoperative values, for each patient. While most patients experienced a decline, some presented unaltered or even increased ccK18 levels. For further analysis, we categorized patients into “Responders” and “Non-Responders”, defining response as a decline in ccK18 levels ≥ 10% one year postoperatively.

Evaluating the individual courses of serum ccK18 levels, not all patients responded to the procedure with a reduction of ccK18 levels (Figure 1D). This observation falls in line with previous biopsy-controlled studies, which reported a worsening or new onset of NAFLD in 5%–20% of patients following bariatric surgery [8]. In our cohort, the reduction of ccK18 was not associated with total body- (*r* = 0.32, *p* = 0.05, Figure 2A) or excessive weight loss (r = 0.12, *p* = 0.462, Figure 2B). On the contrary, a rather modest weight loss of 20% was sufficient to induce the regression of ccK18 levels in some patients. To further investigate this issue, we categorized patients into two groups: A decrease in ccK18 levels by 10% or more one year after surgery when compared to preoperative values was considered a response to treatment. Out of the 39 patients analyzed, 30 met this criterium (Figure 2C). We compared those to the remaining nine patients, which were considered non-responders, to investigate whether the addition of ccK18 levels to a clinical follow-up including routine laboratory parameters could predict the eventual outcome. The results are summarized in Table 2. Preoperative serum triglyceride levels were greater in patients showing a response to bariatric surgery (+42.33 mg/dl, 95% CI 1.4–83.25 mg/dl, *p* = 0.036). In the one-year follow-up, non-responders presented with significantly higher mean GGT (+44.3 U/l, 95% CI 21.21–67.39 U/l, *p* < 0.001).

### 3.5. The Expression of Adipokines and Cytokines Distinguished Responders and Non-Responders

**Figure 3:**
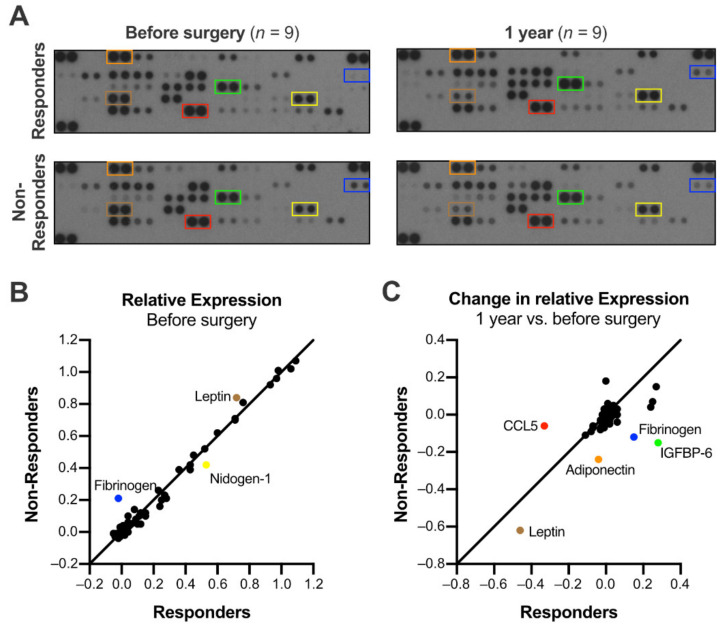
Distinct adipokine expression in responders and non-responders. (**A**) High resolution scans of the original arrays. Pooled serum samples of nine patients per group were subjected to adipokine arrays before and one year after surgery. (**B**) The analysis of the expression relative to positive control dots revealed a greater expression of fibrinogen and leptin in non-responders; nidogen-1 was less abundant in this group. (**C**) One year after surgery, fibrinogen and insulin-like growth factor binding protein 6 (IGFBP-6) expression increased in the response group but decreased in non-responders. Leptin also showed a greater decrease in non-responders. Adiponectin–unchanged in its expression in the response group–decreased by 23.9% in non-responders during the one-year follow up. Contrarily, CCL5 remained stable in non-responders but decreased in patients responding to the bariatric surgery. Correspondingly colored boxes in (**A**) label the protein’s positions on the arrays.

Next, we aimed to evaluate possible differences in the molecular signatures of adipokines and cytokines between responders and non-responders. Before surgery, non-responders showed a strong expression of fibrinogen, while it was hardly detectable in the response group (Figure 3A,B). Non-responders, furthermore, presented with a 14% higher leptin expression and a reduced level of nidogen-1 (−25.6%). Repeated adipokine arrays demonstrated a distinct course of fibrinogen and insulin-like growth factor binding protein 6 (IGFBP-6) expression in responders and non-responders (Figure 3C): both increased in the response group one year after surgery but decreased in non-responders.

Leptin showed a greater decrease in non-responders, leading to a comparable expression one year after surgery. Adiponectin was unchanged in its expression in the response group. In non-responders, however, the expression decreased by 23.9% in the one-year follow up. Finally, cytokine arrays indicated a decreased preoperative expression of CXCL12, plasminogen activator inhibitor 1 (PAI-1), and macrophage migration inhibitory factor (MIF) in non-responders (Figure S3).

## 4. Discussion

The present study elucidates the natural history of ccK18 levels and demonstrates its feasibility in the hepatic follow-up of a cohort undergoing Roux-en-Y gastric bypass. To our knowledge, this is the largest single-center study published to date in which all bariatric procedures were performed by the same surgeon. A significant decline in ccK18 levels was observed six months after surgery, falling in line with a previous report [23].

The high baseline levels of ccK18 and their marked decrease, following the surgery emphasize the extent of liver disease in our bariatric surgery cohort. Although no patient had been diagnosed with a chronic liver disease, the presence of NAFLD was likely in most subjects. According to the NALFD fibrosis score, advanced fibrosis was present in approximately 21% of patients preoperatively. The extent of ccK18 fragments and the high levels of established scores, such as the NFS, indicated progressed disease in some patients. The vigorous examination and thoughtful application of non-invasive biomarkers will be crucial to improve diagnosis, surveillance, and timely therapy of NAFLD in these patients.

Several authors stressed the imperfections of ccK18 as a biomarker for NAFLD. While combinations of liver stiffness measurements and other biomarkers seem promising [25,37], the inherent diagnostic accuracy of ccK18 has often been considered modest at best [38,39]. Furthermore, ideal ccK18 cut-offs are yet to be identified, as Kwok et al. accurately pointed out [28]. On the one hand, the approach presented herein addressed this issue by assessing the individual changes in ccK18 levels over time. Since the release of ccK18 reflects hepatocellular apoptosis as one of the underlying disease mechanisms, it seems especially well-suited to represent the spectrum of metabolism-associated liver disease, rather than imitating histological classifications [23,24,25]. In view of the multiple, short term, and close-knit checks before and after surgery, histological confirmation of the steatosis and fibrosis was not considered. Vuppalanchi et al., moreover, demonstrated that the decrease in ccK18 was correlated with histologic improvement of NAFLD [31]. On the other hand, in an unfiltered cohort without prior evaluation of NAFLD, this approach bears the risk of misinterpreting the lack of response in patients without serious liver disease in the first place. To this end, six out of our nine patients considered non-responders in this study presented with baseline ccK18 levels <200 U/l. In contrast, 13/30 patients, who responded to the intervention, also exhibited such ccK18 levels, and there was no significant difference in the preoperative ccK18 levels between the groups.

Consecutive ccK18 measurements revealed non-responders to bariatric surgery, which showed a decline <10% in ccK18 one year postoperatively when compared to baseline levels, in a similar magnitude as reported previously [8]. Great efforts have been made to reduce heterogeneity in our cohort: all patients were included in a structured, single center treatment regimen including a Roux-en-Y gastric bypass procedure performed by a single experienced surgeon. Yet responses were highly inconsistent. Lacking reduction in waist circumference, higher glucose levels, and insulin resistance have been postulated as possible modes of action [40,41,42]. However, neither the extent of weight loss nor HbA1c values significantly differed between responders and non-responders in our cohort. Further studies are needed to gain a comprehensive understanding of the underlying mechanisms. While the addition of ccK18 to a standard laboratory panel enabled the monitoring of the response to bariatric surgery, it did not increase its performance in predicting the eventual outcome.

However, the differences seen in the expression of adipokines imply promising perspectives. Fibrinogen, for instance, showed a greater expression in non-responders. In these, C-reactive protein was also increased in tendency, indicating a possible role of systemic inflammation. On the contrary, fibrinogen expression increased in responders, while it decreased in non-responders. The use of fibrinogen as a marker of systemic inflammation might also be confounded by the various changes in hemostasis, occurring post bariatric surgery [43]. Leptin was also overexpressed in non-responders. Although there have been conflicting results, a recent meta-analysis found higher circulating leptin levels to be associated with the severity of NAFLD, providing a possible explanation for the distinct course of disease in non-responders [44]. While the expression of adiponectin is comparable among the groups preoperatively, it decreased in non-responders. Adiponectin has been reported to negatively correlate with insulin resistance, visceral fat, advanced fibrosis, and the development of NASH [45,46,47,48]. Shorter intervals of adiponectin measurements might elucidate its potential to predict the response to bariatric surgery in future studies.

In summary, we demonstrated the successful clinical application of a ccK18-based follow-up to monitor the progression of liver disease in a bariatric surgery cohort. The use of non-invasive measures will be inevitable to establish a widespread application of NAFLD surveillance. We renounced histology in favor of close-knit non-invasive controls, as ccK18 has often been correlated with histological findings. The present study facilitates this development by clarifying the natural course of ccK18 in the first year post-bariatric surgery. To fully implement ccK18 in clinical practice, future studies investigating the influence of comorbidities and medication use will be necessary. Furthermore, a focus on the capability of biomarkers to predict outcomes in a subset of patients, rather than predicting the results of the imperfect gold standard liver biopsy, could provide new insights in this rapidly evolving field.

## Figures and Tables

**Table 1:** Baseline parameters of the full cohort before and one year after surgery.

	Preoperative (*n* = 39)	1 Year (*n* = 39)	*p* (Adjusted)
Demographic			
Age (year)	39.44 (23 to 60)		
Female sex	35 (90%)		
Anthropometric			
BMI (kg/m^2^)	51.94 (41.56 to 61.85)	32.64 (17.88 to 54.37)	<0.001
Body weight (kg)	146.54 (111.7 to 190.5)	91.84 (61.3 to 125)	<0.001
Total Body Weight Loss (%)		36.87 (17.88 to 54.37)	
Excess Weight Loss ^†^ (%)		71.89 (38.64 to 105.29)	
Metabolism			
HbA1c (%)	6.19 (4.7 to 9.6)	5.29 (4.5 to 6.7)	<0.001
Diabetes mellitus	10 (31%)	2 (5%)	
LDL cholesterol (mg/dl)	129.65 (53 to 233)	91.92 (20 to 153)	<0.001
HDL cholesterol (mg/dl)	46.32 (27 to 87)	50.79 (17 to 95)	0.021
Serum triglycerides (mg/dl)	173.12 (58 to 751)	88.1 (44 to 253)	<0.001
CRP (mg/l)	17.72 (2.09 to 146.61)	1.87 (0.5 to 14.6)	0.004
Liver-related			
Log ccK18 (U/l)	2.37 (2.01 to 3.17)	2.09 (1.64 to 2.62)	<0.001
ccK18 > 200 U/l	20 (51%)	5 (13%)	
ALT (U/l)	41.03 (11 to 126)	36.15 (10 to 186)	0.914
AST (U/l)	31.15 (10 to 136)	23.44 (8 to 137)	0.285
Alkaline Phosphatase (U/l)	77.44 (48 to 114)	82.74 (43 to 270)	0.836
GGT (U/l)	41 (9 to 162)	23.26 (6 to 279)	0.280
Bilirubin (mg/dl)	0.49 (0.3 to 1)	0.58 (0.2 to 1.5)	0.024
Albumine (g/dl)	4.29 (3.61 to 5.1)	4.41 (3.92 to 5)	0.213
Significant fibrosis?			
NFS	−0.24 (−3.01 to 2.78)	−2.36 (−5.44 to 0.4)	<0.001
NFS > 0.676	8 (21%)	0	
APRI	0.29 (0.06 to 0.99)	0.24 (0.05 to 1.25)	0.576
APRI > 0.7	2 (5%)	1 (3%)	
FIB-4	0.71 (0.23 to 1.67)	0.63 (0.21 to 1.51)	0.311
FIB-4 > 3.25	0	0	

Data are presented as Mean (range) or *n* (%) ^†^ Excess weight was calculated relative to BMI = 25. BMI, Body Mass Index; LDL, Low Density Lipoprotein; HDL, High Density Lipoprotein; CRP, C-reactive protein; ccK18, caspase-cleaved keratin 18 (M30); ALT, alanine-aminotransferase; AST, aspartate-aminotransferase; GGT, gamma-glutamyl-transferase; NFS, NAFLD fibrosis score; APRI, aspartate-aminotransferase to platelet ratio index.

**Table 2:** Comparison of responders and non-responders before and one year after surgery.

	Preoperative			1 Year		
	Responders (*n* = 30)	Non-Responders (*n* = 9)	*p* (Adjusted)	Responders (*n* = 30)	Non-Responders (*n* = 9)	*p* (Adjusted)
Demographic						
Age (year)	39.1 (23 to 60)	40.56 (27 to 51)	>0.999			
Female sex	27 (90%)	8 (89%)				
Anthropometric						
BMI (kg/m^2^)	51.4 (41.56 to 61.85)	53.72 (44.92 to 59.88)	>0.999	32.89 (25.4 to 42.52)	31.81 (23.95 to 40.15)	>0.999
Body weight (kg)	145.51 (111.7 to 190.5)	149.98 (115 to 183)	>0.999	92.79 (73.4 to 125)	88.68 (61.3 to 108)	>0.999
Metabolism						
Diabetes mellitus	8 (33%)	2 (25%)		1 (3%)	1 (11%)	
LDL cholesterol (mg/dl)	128.15 (53 to 233)	134.5 (90 to 165)	>0.999	90.63 (20 to 145)	96.22 (65 to 153)	>0.999
HDL cholesterol (mg/dl)	44.5 (27 to 71)	52.25 (31 to 87)	>0.999	49.3 (17 to 83)	55.78 (38 to 95)	>0.999
Serum triglycerides (mg/dl)	183.08 (58 to 751)	140.75 (98 to 189)	0.036	90.53 (44 to 253)	80 (44 to 120)	0.962
CRP (mg/l)	15.25 (2.09 to 146.61)	25.94 (8.12 to 110.89)	>0.999	1.26 (0.5 to 7.91)	3.9 (0.5 to 14.6)	>0.999
Liver-related						
Log ccK18 (U/l)	2.4 (2.01 to 3.17)	2.27 (2.13 to 2.53)	>0.999	2.02 (1.64 to 2.28)	2.34 (2.21 to 2.62)	>0.999
ccK18 > 200 U/l	17 (57%)	3 (33%)		0	5 (56%)	
ALT (U/l)	45.5 (11 to 126)	26.11 (13 to 43)	0.921	34.47 (10 to 186)	41.78 (10 to 102)	>0.999
AST (U/l)	34.13 (10 to 136)	21.22 (12 to 30)	>0.999	22.87 (8 to 137)	25.33 (12 to 42)	>0.999
Alkaline Phosphatase (U/l)	76.87 (48 to 114)	79.33 (50 to 114)	>0.999	78.27 (43 to 122)	97.67 (60 to 270)	0.194
GGT (U/l)	45.33 (9 to 162)	26.56 (11 to 56)	0.939	13.03 (6 to 40)	57.33 (10 to 279)	< 0.001
Bilirubin (mg/dl)	0.49 (0.3 to 1)	0.5 (0.3 to 0.7)	>0.999	0.59 (0.2 to 1.5)	0.54 (0.3 to 0.9)	>0.999
Albumine (g/dl)	4.35 (3.8 to 5.1)	4.09 (3.6 to 4.6)	>0.999	4.47 (4.03 to 5)	4.24 (3.92 to 4.5)	>0.999
Significant fibrosis?						
NFS	−0.27	−0.164	>0.999	−2.21 (−4.68 to 0.4)	−2.87 (−5.44 to −0.44)	>0.999
NFS > 0.676	6 (20%)	2 (22%)		0	0	
APRI	0.318	0.187	>0.999	0.24 (0.05 to 1.25)	0.23 (0.09 to 0.48)	>0.999
APRI > 0.7	2 (7%)	0		1 (3%)	0	
FIB-4	0.74	0.594	>0.999	0.64 (0.21 to 1.51)	0.59 (0.36 to 1.33)	>0.999
FIB-4 > 3.25	0	0		0	0	

Data are given as Mean or Median (range) or *n* (%) LDL, Low Density Lipoprotein; HDL, High Density Lipoprotein; CRP, C-reactive protein; ccK18, caspase-cleaved Keratin 18 (M30); ALT, alanine-aminotransferase; AST, aspartate-aminotransferase; GGT, gamma-glutamyl-transferase; NFS, NAFLD fibrosis score; APRI, aspartate-aminotransferase to platelet ratio index.

## Data Availability

The data presented in this study are available on request from the corresponding author.

## Supplementary Materials

The following are available online at https://www.mdpi.com/2077-0383/10/6/1233/s1, Figure S1: Fasting is not required to obtain reliable ccK18 serum levels, Figure S2: Natural history of parameters of liver injury following bariatric surgery, Figure S3: Cytokine expression in responders and non-responders.

## References

[1] Matteoni C.A., Younossi Z.M., Gramlich T., Boparai N., Liu Y.C., McCullough A.J. (1999). Nonalcoholic fatty liver disease: A spectrum of clinical and pathological severity. Gastroenterology.

[2] Vernon G., Baranova A., Younossi Z.M. (2011). Systematic review: The epidemiology and natural history of non-alcoholic fatty liver disease and non-alcoholic steatohepatitis in adults. Aliment. Pharmacol. Ther..

[3] Younossi Z., Tacke F., Arrese M., Chander Sharma B., Mostafa I., Bugianesi E., Wai-Sun Wong V., Yilmaz Y., George J., Fan J. (2019). Global Perspectives on Nonalcoholic Fatty Liver Disease and Nonalcoholic Steatohepatitis. Hepatology.

[4] Younossi Z.M., Marchesini G., Pinto-Cortez H., Petta S. (2019). Epidemiology of Nonalcoholic Fatty Liver Disease and Nonalcoholic Steatohepatitis: Implications for Liver Transplantation. Transplantation.

[5] Friedman S.L., Neuschwander-Tetri B.A., Rinella M., Sanyal A.J. (2018). Mechanisms of NAFLD development and therapeutic strategies. Nat. Med..

[6] De Ridder R.J., Schoon E.J., Smulders J.F., van Hout G.C., Stockbrugger R.W., Koek G.H. (2007). Review article: Non-alcoholic fatty liver disease in morbidly obese patients and the effect of bariatric surgery. Aliment. Pharmacol. Ther..

[7] Mummadi R.R., Kasturi K.S., Chennareddygari S., Sood G.K. (2008). Effect of bariatric surgery on nonalcoholic fatty liver disease: Systematic review and meta-analysis. Clin. Gastroenterol. Hepatol..

[8] Lee Y., Doumouras A.G., Yu J., Brar K., Banfield L., Gmora S., Anvari M., Hong D. (2019). Complete Resolution of Nonalcoholic Fatty Liver Disease After Bariatric Surgery: A Systematic Review and Meta-analysis. Clin. Gastroenterol. Hepatol..

[9] Roeb E., Steffen H.M., Bantel H., Baumann U., Canbay A., Demir M., Drebber U., Geier A., Hampe J., Hellerbrand C. (2015). S2k Guideline non-alcoholic fatty liver disease. Z. Gastroenterol..

[10] European Association for the Study of the Liver, European Association for the Study of Diabetes (2016). Clinical Practice Guidelines for the management of non-alcoholic fatty liver disease. J. Hepatol..

[11] Chalasani N., Younossi Z., Lavine J.E., Charlton M., Cusi K., Rinella M., Harrison S.A., Brunt E.M., Sanyal A.J. (2018). The diagnosis and management of nonalcoholic fatty liver disease: Practice guidance from the American Association for the Study of Liver Diseases. Hepatology.

[12] Chitturi S., Wong V.W., Chan W.K., Wong G.L., Wong S.K., Sollano J., Ni Y.H., Liu C.J., Lin Y.C., Lesmana L.A. (2018). The Asia-Pacific Working Party on Non-alcoholic Fatty Liver Disease guidelines 2017-Part 2: Management and special groups. J. Gastroenterol. Hepatol..

[13] Kroh M., Liu R., Chand B. (2007). Laparoscopic bariatric surgery: What else are we uncovering? Liver pathology and preoperative indicators of advanced liver disease in morbidly obese patients. Surg. Endosc..

[14] Machado M., Marques-Vidal P., Cortez-Pinto H. (2006). Hepatic histology in obese patients undergoing bariatric surgery. J. Hepatol..

[15] Harnois F., Msika S., Sabate J.M., Mechler C., Jouet P., Barge J., Coffin B. (2006). Prevalence and predictive factors of non-alcoholic steatohepatitis (NASH) in morbidly obese patients undergoing bariatric surgery. Obes. Surg..

[16] Caulin C., Salvesen G.S., Oshima R.G. (1997). Caspase cleavage of keratin 18 and reorganization of intermediate filaments during epithelial cell apoptosis. J. Cell Biol..

[17] Bantel H., Ruck P., Gregor M., Schulze-Osthoff K. (2001). Detection of elevated caspase activation and early apoptosis in liver diseases. Eur. J. Cell Biol..

[18] Bantel H., Lugering A., Heidemann J., Volkmann X., Poremba C., Strassburg C.P., Manns M.P., Schulze-Osthoff K. (2004). Detection of apoptotic caspase activation in sera from patients with chronic HCV infection is associated with fibrotic liver injury. Hepatology.

[19] Kronenberger B., Wagner M., Herrmann E., Mihm U., Piiper A., Sarrazin C., Zeuzem S. (2005). Apoptotic cytokeratin 18 neoepitopes in serum of patients with chronic hepatitis C. J. Viral. Hepat..

[20] Feldstein A.E., Canbay A., Angulo P., Taniai M., Burgart L.J., Lindor K.D., Gores G.J. (2003). Hepatocyte apoptosis and fas expression are prominent features of human nonalcoholic steatohepatitis. Gastroenterology.

[21] Wieckowska A., Zein N.N., Yerian L.M., Lopez A.R., McCullough A.J., Feldstein A.E. (2006). In vivo assessment of liver cell apoptosis as a novel biomarker of disease severity in nonalcoholic fatty liver disease. Hepatology.

[22] Younossi Z.M., Jarrar M., Nugent C., Randhawa M., Afendy M., Stepanova M., Rafiq N., Goodman Z., Chandhoke V., Baranova A. (2008). A novel diagnostic biomarker panel for obesity-related nonalcoholic steatohepatitis (NASH). Obes. Surg..

[23] Diab D.L., Yerian L., Schauer P., Kashyap S.R., Lopez R., Hazen S.L., Feldstein A.E. (2008). Cytokeratin 18 fragment levels as a noninvasive biomarker for nonalcoholic steatohepatitis in bariatric surgery patients. Clin. Gastroenterol. Hepatol..

[24] Feldstein A.E., Wieckowska A., Lopez A.R., Liu Y.C., Zein N.N., McCullough A.J. (2009). Cytokeratin-18 fragment levels as noninvasive biomarkers for nonalcoholic steatohepatitis: A multicenter validation study. Hepatology.

[25] Liebig S., Stoeckmann N., Geier A., Rau M., Schattenberg J.M., Bahr M.J., Manns M.P., Jaeckel E., Schulze-Osthoff K., Bantel H. (2019). Multicenter Validation Study of a Diagnostic Algorithm to Detect NASH and Fibrosis in NAFLD Patients With Low NAFLD Fibrosis Score or Liver Stiffness. Clin. Transl. Gastroenterol..

[26] Bantel H., John K., Schulze-Osthoff K. (2014). Robust detection of liver steatosis and staging of NAFLD by an improved ELISA for serum cytokeratin-18 fragments. Am. J. Gastroenterol..

[27] Joka D., Wahl K., Moeller S., Schlue J., Vaske B., Bahr M.J., Manns M.P., Schulze-Osthoff K., Bantel H. (2012). Prospective biopsy-controlled evaluation of cell death biomarkers for prediction of liver fibrosis and nonalcoholic steatohepatitis. Hepatology.

[28] Kwok R., Tse Y.K., Wong G.L., Ha Y., Lee A.U., Ngu M.C., Chan H.L., Wong V.W. (2014). Systematic review with meta-analysis: Non-invasive assessment of non-alcoholic fatty liver disease--the role of transient elastography and plasma cytokeratin-18 fragments. Aliment. Pharmacol. Ther..

[29] Musso G., Gambino R., Cassader M., Pagano G. (2011). Meta-analysis: Natural history of non-alcoholic fatty liver disease (NAFLD) and diagnostic accuracy of non-invasive tests for liver disease severity. Ann. Med..

[30] Safarian M., Mohammadpour S., Shafiee M., Ganji A., Soleimani A., Nematy M., Bahari A. (2019). Effect of diet-induced weight loss on cytokeratin-18 levels in overweight and obese patients with liver fibrosis. Diabetes Metab. Syndr..

[31] Vuppalanchi R., Jain A.K., Deppe R., Yates K., Comerford M., Masuoka H.C., Neuschwander-Tetri B.A., Loomba R., Brunt E.M., Kleiner D.E. (2014). Relationship between changes in serum levels of keratin 18 and changes in liver histology in children and adults with nonalcoholic fatty liver disease. Clin. Gastroenterol. Hepatol..

[32] Tsutsui M., Tanaka N., Kawakubo M., Sheena Y., Horiuchi A., Komatsu M., Nagaya T., Joshita S., Umemura T., Ichijo T. (2010). Serum fragmented cytokeratin 18 levels reflect the histologic activity score of nonalcoholic fatty liver disease more accurately than serum alanine aminotransferase levels. J. Clin. Gastroenterol..

[33] Hempel F., Roderfeld M., Savai R., Sydykov A., Irungbam K., Schermuly R., Voswinckel R., Kohler K., Churin Y., Kiss L. (2019). Depletion of Bone Marrow-Derived Fibrocytes Attenuates TAA-Induced Liver Fibrosis in Mice. Cells.

[34] Lin Z.H., Xin Y.N., Dong Q.J., Wang Q., Jiang X.J., Zhan S.H., Sun Y., Xuan S.Y. (2011). Performance of the aspartate aminotransferase-to-platelet ratio index for the staging of hepatitis C-related fibrosis: An updated meta-analysis. Hepatology.

[35] Sterling R.K., Lissen E., Clumeck N., Sola R., Correa M.C., Montaner J., Sulkowski M.S., Torriani F.J., Dieterich D.T., Thomas D.L. (2006). Development of a simple noninvasive index to predict significant fibrosis in patients with HIV/HCV coinfection. Hepatology.

[36] Angulo P., Hui J.M., Marchesini G., Bugianesi E., George J., Farrell G.C., Enders F., Saksena S., Burt A.D., Bida J.P. (2007). The NAFLD fibrosis score: A noninvasive system that identifies liver fibrosis in patients with NAFLD. Hepatology.

[37] He L., Deng L., Zhang Q., Guo J., Zhou J., Song W., Yuan F. (2017). Diagnostic Value of CK-18, FGF-21, and Related Biomarker Panel in Nonalcoholic Fatty Liver Disease: A Systematic Review and Meta-Analysis. BioMed Res. Int..

[38] Cusi K., Chang Z., Harrison S., Lomonaco R., Bril F., Orsak B., Ortiz-Lopez C., Hecht J., Feldstein A.E., Webb A. (2014). Limited value of plasma cytokeratin-18 as a biomarker for NASH and fibrosis in patients with non-alcoholic fatty liver disease. J. Hepatol..

[39] Wong V.W., Adams L.A., de Ledinghen V., Wong G.L., Sookoian S. (2018). Noninvasive biomarkers in NAFLD and NASH—Current progress and future promise. Nat. Rev. Gastroenterol. Hepatol..

[40] Schwenger K.J.P., Fischer S.E., Jackson T., Okrainec A., Allard J.P. (2018). In nonalcoholic fatty liver disease, Roux-en-Y gastric bypass improves liver histology while persistent disease is associated with lower improvements in waist circumference and glycemic control. Surg. Obes. Relat. Dis..

[41] Mathurin P., Gonzalez F., Kerdraon O., Leteurtre E., Arnalsteen L., Hollebecque A., Louvet A., Dharancy S., Cocq P., Jany T. (2006). The evolution of severe steatosis after bariatric surgery is related to insulin resistance. Gastroenterology.

[42] Mathurin P., Hollebecque A., Arnalsteen L., Buob D., Leteurtre E., Caiazzo R., Pigeyre M., Verkindt H., Dharancy S., Louvet A. (2009). Prospective study of the long-term effects of bariatric surgery on liver injury in patients without advanced disease. Gastroenterology.

[43] Tuovila M., Erkinaro T., Koivukangas V., Savolainen E.R., Laurila P., Ohtonen P., Ala-Kokko T. (2018). Thromboelastography Values Remain Hypercoagulative 6 Months After Obesity Surgery: A Pilot Study. Obes. Surg..

[44] Polyzos S.A., Aronis K.N., Kountouras J., Raptis D.D., Vasiloglou M.F., Mantzoros C.S. (2016). Circulating leptin in non-alcoholic fatty liver disease: A systematic review and meta-analysis. Diabetologia.

[45] Jarrar M.H., Baranova A., Collantes R., Ranard B., Stepanova M., Bennett C., Fang Y., Elariny H., Goodman Z., Chandhoke V. (2008). Adipokines and cytokines in non-alcoholic fatty liver disease. Aliment. Pharmacol. Ther..

[46] Polyzos S.A., Toulis K.A., Goulis D.G., Zavos C., Kountouras J. (2011). Serum total adiponectin in nonalcoholic fatty liver disease: A systematic review and meta-analysis. Metabolism.

[47] Balmer M.L., Joneli J., Schoepfer A., Stickel F., Thormann W., Dufour J.F. (2010). Significance of serum adiponectin levels in patients with chronic liver disease. Clin. Sci..

[48] Boutari C., Mantzoros C.S. (2020). Adiponectin and leptin in the diagnosis and therapy of NAFLD. Metabolism.

