# Supplementary material for: Caspase-Cleaved Keratin 18 Measurements Identified Ongoing Liver Injury after Bariatric Surgery
Source: J Clin Med. 2021 Mar 16;10(6):1233. doi: 10.3390/jcm10061233 (PMC8002276; doi:10.3390/jcm10061233)
Supplement: Supplementary file 1 [file jcm-10-01233-s001.pdf]

Journal of Clinical Medicine

Supplementary material

Caspase-cleaved keratin 18 measurements identified ongoing liver injury after bariatric surgery

Felix Hempel [1,†], Martin Roderfeld [1,†], Lucas J. Müntnich [1], Jens Albrecht [2,3], Ziya Oruc [2], Borros Arneth [4,5], Thomas Karrasch [6], Jörn Pons-Kühnemann [7], Winfried Padberg [2], Harald Renz [4,5], Andreas Schäffler [6] and Elke Roeb [1,*]

[1] Department of Gastroenterology, Justus Liebig University, Giessen, Germany; felix.hempel@med.jlug.de (F.H.); martin.roderfeld@innere.med.uni-giessen.de (M.R.); lucas.j.muentnich@med.uni-giessen.de (L.J.M.)
[2] Department of General, Visceral, Thoracic, Transplantation, and Pediatric Surgery, Justus Liebig University, Giessen, Germany; z.oruc@web.de (Z.O.); winfried.padberg@chiru.med.uni-giessen.de (W.P.)
[3] Department for Bariatric Surgery, Asklepios Hospital, Lich, Germany; j.albrecht@azhm.de
[4] Institute of Laboratory Medicine, Pathobiochemistry and Molecular Diagnostics, Justus Liebig University, Giessen, Germany; borros.arneth@klinchemie.med.uni-giessen.de (B.A.); renzh@med.uni-marburg.de (H.R.)
[5] Member of the German Lung Center (DZL) and the Universities of Giessen and Marburg Lung Center (UG-MLC)
[6] Department of Internal Medicine III, Justus Liebig University, Giessen, Germany; thomas.karrasch@innere.med.uni-giessen.de (T.K.); andreas.schaeffler@innere.med.uni-giessen.de (A.S.)
[7] Institute of Medical Informatics, Justus Liebig University, Giessen, Germany; Joern.Pons@informatik.med.uni-giessen.de
* Correspondence: elke.roeb@innere.med.uni-giessen.de; Tel.: +49 (0) 641 98542338
† These authors contributed equally.

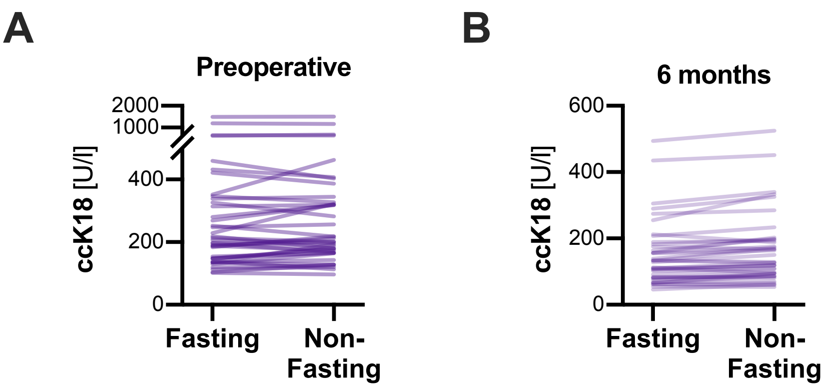

Supplementary Figure S1. Fasting is not required to obtain reliable ccK18 serum levels. Blood samples were drawn from all 39 patients **(A)** before and **(B)** six months after the surgery – once fasting, once after a standard meal. The coefficient of variation, caused by the additional blood sample, was lower than the interassay variability proposed by the manufacturer, indicating no substantial influence of the fasting state.

Copyright: © 2021 by the authors. Submitted for possible open access publication under the terms and conditions of the Creative Commons Attribution (CC BY) license (http://creativecommons.org/licenses/by/4.0/).

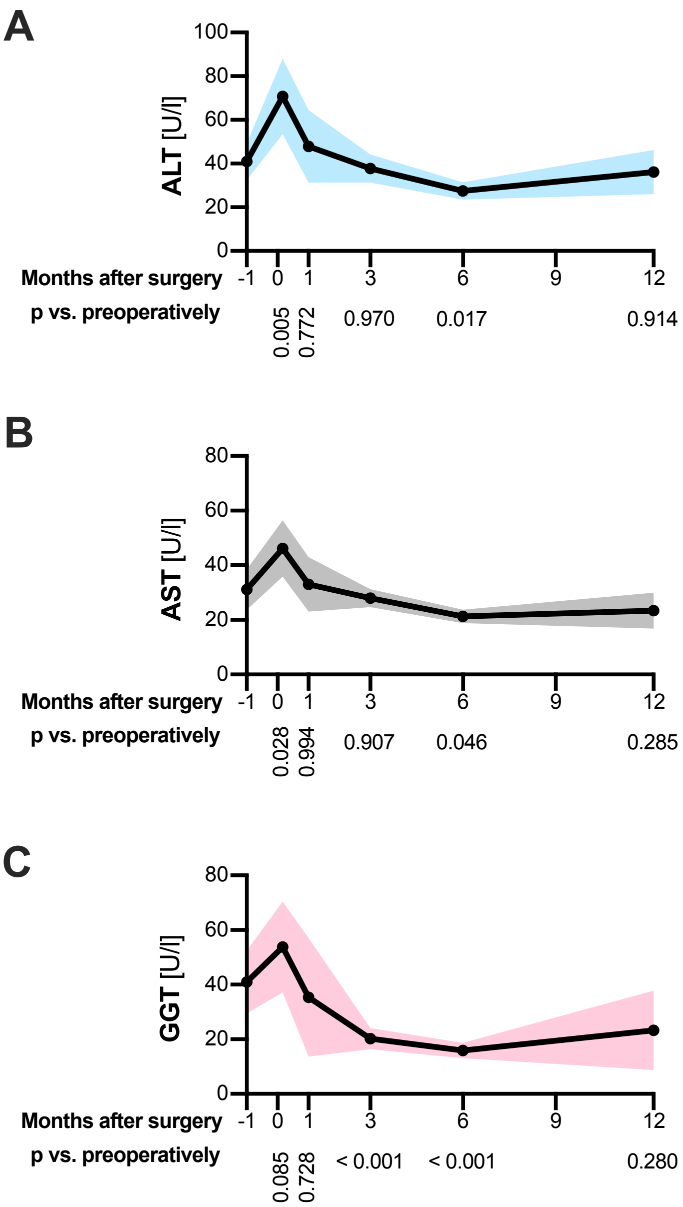

Supplementary Figure S2. Natural history of parameters of liver injury following bariatric surgery. The course of **(A)** alanine-aminotransferase (ALT), **(B)** aspartate-aminotransferase (AST), and **(C)** gamma-glutamyltransferase (GGT) was distinct from that of caspase-cleaved keratin 18 (Figure 1C). All three parameters showed a transient increase subsequent to surgery. However, the levels decrease one month postoperatively. Interestingly, there is a significant decrease in ALT, AST, and GGT six months but not 12 months after surgery. Mean (line) and 95% confidence interval (colored area) are shown. A fitted mixed-effects model was applied.

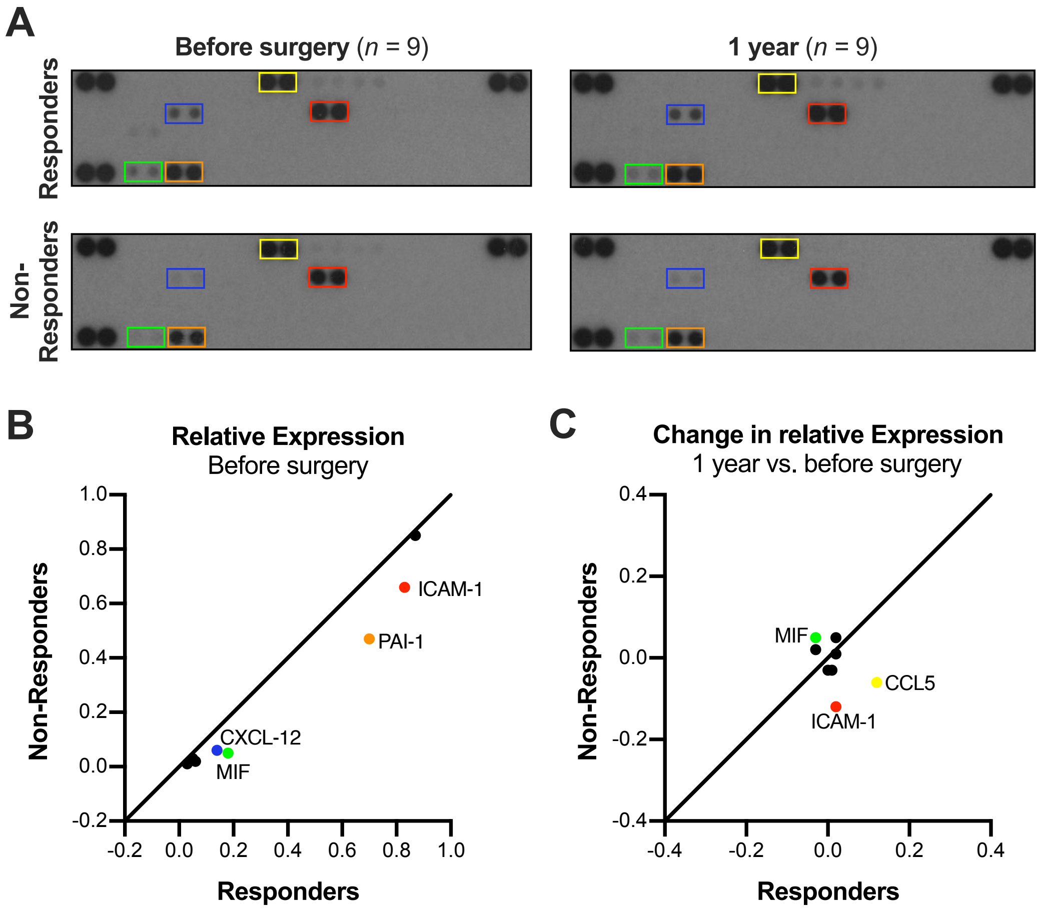

Supplementary Figure S3. Cytokine expression in responders and non-responders. **(A)** High resolution scans of the original arrays. Pooled serum samples of 9 patients per group were subjected to cytokine arrays before and one year after surgery. **(B)** Preoperatively, the expression of CXCL12, plasminogen activator inhibitor 1 (PAI-1), and macrophage migration inhibitory factor (MIF) was lower in non-responders. **(C)** One year after gastric bypass, the expression of intercellular adhesion molecule 1 (ICAM-1) decreased in non-responders, while remaining unchanged in responders. CCL5 is more abundant in responders one year postoperatively but less expressed in non-responders. Correspondingly colored boxes in **(A)** label the protein's positions on the arrays.
